# N-Acetyl-Aspartyl Glutamic Acid (NAAGA)-Based Eye Drops for Contact Lens Wearers with Dry Eye Symptoms and Discomfort

**DOI:** 10.3390/vision10010001

**Published:** 2025-12-22

**Authors:** Ioanna Misheva, Vesselin Daskalov, Dimitar Dzhelebov, Kalina Ilieva, Ralitsa Kermedchieva, Malina Topchiyska, Petar Yanev, Christina Grupcheva

**Affiliations:** 1 Medical Center for Eye Health Focus, 123 Bylgarska Morava Str., 1303 Sofia, Bulgaria; ipm_md@abv.bg (I.M.); dr.kalina.ilieva@abv.bg (K.I.); 2 Specialized Ophthalmological Hospital for Active Treatment Pentagram, 109-111, Vranya Str., 1309 Sofia, Bulgaria; ves.daskalov61@gmail.com (V.D.); rkermedchieva@yahoo.com (R.K.); 3 Medical Center Vereya, 4, Kenaly Str. Stara, 6000 Zagora, Bulgaria; dzhelebov@gmail.com; 4 Medical Center Oxycom Complex Izgrev, Bl. 28, Entr. A, Ground Floor, 8000 Burgas, Bulgaria; mdjokowa@yahoo.com; 5 Asmp Ob–Ip Glm Eood, 28 Han Presian Street, 4700 Smolyan, Bulgaria; yanev.petar@yahoo.com; 6 Institute for New Technologies in Ophthalmology (INTO), Seliolu1A, 9002 Varna, Bulgaria

**Keywords:** contact lens, dry eye disease, lubricating eye drops, anti-inflammatory, bioprotectant

## Abstract

The aim of this study was to evaluate the performance and safety of T2769 (Thealoz^®^ Total), a preservative-free eye drop combining 0.15% sodium hyaluronate, 3% trehalose, and 2.45% N-acetylaspartyl-glutamate (NAAGA), in contact lens wearers with dry eye symptoms and discomfort. This prospective, single-arm investigation enrolled 34 adult contact lens wearers with Ocular Surface Disease Index (OSDI) scores ≥ 18 and Contact Lens Dry Eye Questionnaire-8 (CLDEQ-8) scores ≥ 12. Patients instilled one drop of T2769 three to six times daily for 36 days. Performance assessments included CLDEQ-8, ocular discomfort and symptoms, OSDI, soothing sensation, and ocular signs. Safety assessments included adverse events (AEs), far BCVA, and ocular tolerance. CLDEQ-8 improved from the baseline at Day 36 (−12.6 ± 5.0; *p* < 0.001) and as early as D15, with similar improvements in ocular discomfort, OSDI, and total symptom score. Soothing sensation was judged important by 79.4% of patients at D36. Ocular surface staining, tear break-up time, and the Schirmer test improved at D15 and D36, while conjunctival hyperaemia improved in 82.4% of patients at D36. Two non-serious treatment-related AEs (photophobia and blurred vision) occurred in one patient. BCVA was unchanged, and tolerance was rated very satisfactory/satisfactory. In conclusion, T2769 was safe and effective for reducing contact lens-associated dry eyes and discomfort.

## 1. Introduction

Dry eye disease (DED) is highly prevalent worldwide, with a considerable humanistic and economic burden [1,2]. It is defined as “a multifactorial, symptomatic disease characterised by a loss of homeostasis of the tear film and/or ocular surface, in which tear film instability and hyperosmolarity, ocular surface inflammation and damage, and neurosensory abnormalities are etiological factors” [2]. Inflammation is considered to contribute to and perpetuate the ocular surface damage and symptom generation in dry eyes. Environmental factors such as contact lenses can amplify this inflammatory response, potentially by thinning the tear film after insertion and increasing friction between the lens and ocular surface, leading to meibomian gland dropout, decreased tear film stability, ocular surface staining, and eyelid wiper epitheliopathy [3]. The use of contact lenses is also widespread, with almost 90% of users preferring soft contact lenses [4]. However, wearing contact lenses is associated with inflammatory events and may cause or exacerbate symptoms and signs of DED [5]. It has been estimated that up to 30% of contact lens wearers experience dry eye symptoms such as ocular grittiness or burning [6], and in several epidemiological studies, the use of contact lenses is considered a risk factor for dry eyes [7]. Ocular dryness in contact lenses wearers commonly refers to contact lens discomfort (CLD) based on symptoms alone [8], and is defined as “a condition characterised by episodic or persistent adverse ocular sensations related to lens wear, either with or without visual disturbance, resulting from reduced compatibility between the contact lens and the ocular environment, which can lead to decreased wearing time and discontinuation of contact lens wear” [9].

CLD management strategies include changes to the contact lens care system, material, design, and replacement schedule, punctal plug insertion, dietary supplementation, and improvement of environment. More advanced approaches such as intense pulsed light combined with low-level light therapy have recently shown benefits in refractory meibomian gland dysfunction [10]. Tear supplements and wetting agents are also widely used, with a preference for preservative-free eye drops due to their favourable safety profile [11]. TFOS DEWS III confirmed that first-line management of DED should focus on methods to replenish, conserve, and stimulate the tear film, with an emphasis on ocular supplements [8]. Lubrication of the ocular surface is the most commonly used first-line treatment for DED, providing symptomatic relief and protecting the ocular surface. Medicated tear supplements combine a lubricating polymer as the primary component with a low-concentration active pharmacological ingredient serving as an ancillary agent to support its therapeutic effect [8]. Various combinations of agents with different mechanisms of action can further improve signs, symptoms, and comfort [12].

In the context of DED management, clinical studies have demonstrated the safety and efficacy of eye drops containing the anti-inflammatory peptide N-acetylaspartyl-glutamate (NAAGA) [13], as well as Thealoz^®^ Duo (Laboratoires Théa, Clermont-Ferrand, France), which contains 0.15% sodium hyaluronate and 3% trehalose [14,15]. Naabak^®^ (NAAGA 4.9%; Laboratoires Théa, Clermont-Ferrand, France) is a well-established anti-inflammatory agent used for the treatment allergic conjunctivitis in its acute or chronic form. The clinical experience with this drug marketed for more than 40 years showed a favourable safety profile. T2769, marketed as Thealoz^®^ Total (Laboratoires Théa, Clermont-Ferrand, France), is a new preservative-free eye drop solution that combines 0.15% sodium hyaluronate and 3% trehalose with 2.45% NAAGA. In patients with moderate to severe DED, T2769 was well-tolerated and led to clinically meaningful improvements in ocular symptoms and signs after 42 days of treatment, with an improvement apparent by 14 days of treatment. By D42, most patients reported no or only mild symptoms and notable improvements in conjunctival hyperaemia, corneal and conjunctival staining, and soothing sensation [16]. The present clinical investigation evaluated the ocular performance and safety of T2769 in contact lens wearers experiencing dry eyes and CLD to determine its clinical benefits in this population.

## 2. Method

This prospective, single-arm clinical investigation was conducted at six sites in Bulgaria (ClinicalTrials.gov Identifier: NCT05931861). Prior to initiation, the clinical investigation plan was approved by a Central Ethics Committee according to regional and local regulatory requirements. The clinical investigation was performed according to the ethical principles of the Declaration of Helsinki and the International Council for Harmonisation guidelines for Good Clinical Practice. Each patient signed an informed consent form prior to inclusion. The clinical investigation took place between August and December 2023.

### 2.1. Patients

Eligible patients were adults (≥18 years) wearing well-fitted contact lenses for at least 6 h per day, 5 days per week, for a minimum of 1 month prior to enrolment, with an Ocular Surface Disease Index (OSDI) score ≥ 18, a Contact Lens Dry Eye Questionnaire-8 (CLDEQ-8) score ≥ 12, and no use of dry eye treatments within 24 h prior to study entry.

The main exclusion criteria were: Far Best-Corrected Visual Acuity (BCVA) ≥ +0.7 LogMAR; severe blepharitis or Meibomian gland dysfunction; palpebral or nasolacrimal disorders; and dry eyes associated with ocular rosacea, pterygium, eyelid malposition, corneal dystrophy, ocular neoplasia, filamentous keratitis, corneal neovascularisation, orbital radiotherapy, cataract, or retinal disease. Patients with a history of severe ocular trauma, ocular infection, ocular inflammation, uveitis, or inflammatory corneal ulcer in the preceding 12 months were also excluded, as were those with ocular allergy that was active or at risk of reactivation, prior refractive surgery, or glaucoma or ocular hypertension requiring treatment. Any condition judged to be incompatible with the study objectives led to exclusion. No restrictions were applied regarding lens material, brand, replacement schedule, or wearing modality, except that orthokeratology contact lenses were excluded.

### 2.2. Study Design

Patients were required to wear contact lenses on a daily basis and remove them at the end of each day. They were instructed to instil one drop of T2769 in each eye, three to six times daily, while wearing their contact lenses, for 36 days. T2769 was initiated immediately after inclusion (no run-in or wash-out was performed). Throughout the study, patients completed a diary and attended the investigational site on Day 1 (D1; Visit 1), Day 15 (D15; Visit 2), and Day 36 (D36; Visit 3) for clinical assessments and diary reviews. At each visit, patients came to the investigation site wearing their contact lenses. After the far BCVA assessment, the lenses were removed for the remaining ocular examinations and reinserted at the end of the visit.

### 2.3. Test Product

T2769 (Laboratoires Théa, Clermont-Ferrand, France) eye drops are preservative-free and contain 0.15% sodium hyaluronate, 3% trehalose, and 2.45% NAAGA, packaged in 12.5 mL multidose bottles. The final pH of the formulation was 7.2 and the osmolality was 330 mosmol/kg.

### 2.4. Performance and Safety Assessments

The primary performance endpoint was the change from baseline (D1) to D36 in the CLDEQ-8 total score, a validated 8-item questionnaire, generating a total score ranging from 1 (minimum discomfort) to 37 (maximum discomfort) [17,18].

The following secondary performance measures were conducted on D1, D15, and D36. Daily contact lens wearing time (hours per day) was recorded by patients.

Ocular discomfort was assessed by the patient using a visual analogue scale (VAS; 0 mm [no discomfort] to 100 mm [maximum discomfort]).

Dry eye symptoms were also evaluated with the OSDI questionnaire [19,20], with scores categorised as normal (≥0 and <13), mild (≥13 and <23), moderate (≥23 and <33), or severe (≥33 and ≤100) [21]. An OSDI score of 18 was considered the diagnostic threshold for DED, with scores also evaluated in classes of <18 and ≥18.20. Patients rated ocular symptoms throughout the day (burning/irritation, stinging/eye pain, itching/pruritus, eye dryness feeling, tearing, and foreign body sensation) for both eyes on a 4-point scale of 0 (absent), 1 (mild, present but not disturbing), 2 (moderate, disturbing, but not limiting daily activities), 3 (severe, very distressing, and interfering with daily activities), with a total score 0–18.

Slit lamp examination included conjunctival hyperaemia (McMonnies 0–5 scale) [22], tear break-up time (TBUT, average of three measures per eye), and ocular staining (Oxford 0–15 scheme). Staining was assessed after instillation of fluorescein Faure 0.5% (SERB SA, Bruxelles, Belgique), with 10× magnification, constant cobalt blue illumination, and a yellow filter. Schirmer test I was performed without anaesthesia (mm/5 min) for each eye. On D15 and D36, patients rated the soothing sensation of T2769 within 5 min after instillation on a 4-point scale (0 = none, 1 = mild, 2 = moderate, and 3 = important), and the investigator evaluated the overall performance (unsatisfactory, not very satisfactory, satisfactory, or very satisfactory).

Treatment compliance was recorded by patients in daily diaries, which were reviewed at each visit. At each visit, new clinically significant ocular and systemic signs or symptoms, or worsening of pre-existing signs or symptoms, were documented and reported as AEs. For each AE, the investigator assessed the causal relationship to T2769. Far BCVA was assessed in each eye on D1 and D36 using the same chart (e.g., a Snellen chart) and the results were analysed after conversion to LogMAR. The investigator and patient evaluated overall ocular tolerance as unsatisfactory, not very satisfactory, satisfactory, or very satisfactory.

### 2.5. Statistical Analyses

The primary performance parameter was the change in CLDEQ-8 total score from baseline (i.e., D1, prior to the first instillation of T2769) to D36. Performance and safety data were analysed using descriptive statistics. For all quantitative endpoints, normality tests were performed. When normally distributed, data are reported as the mean change from baseline (mean, SD; 95% CI; *p*-value); when normality was not met, they are presented as median (min; max). Statistical comparisons were performed for quantitative parameters using paired *t*-tests (or Signed rank tests in cases with violation of the assumption of normality) and for qualitative variables using Signed rank tests.

Statistical tests were 2-sided, and the acceptable level of significance was set at 5%. Pairwise comparisons for endpoints were exploratory. All *p*-values were nominal with no adjustment for multiplicity.

Eyes were considered eligible if they met all inclusion criteria and no ophthalmic exclusion criteria. For patients with two eligible eyes, the study eye was selected based on the worse baseline Oxford total score. If scores were equal, the eye with the worse baseline Schirmer score was chosen, followed by the worse baseline TBUT score. If no differences were present, the right eye was the study eye.

Performance data are presented for the Full Analysis Set (FAS), defined as all patients who received at least one instillation of T2769 and had at least one baseline and one post-baseline performance assessment), and safety data are presented for the Safety Set, including all patients who received at least one instillation of T2769.

Data analyses were performed using SAS Studio 5.2 under SAS Viya 3.5 (SAS Institute Inc., Cary, NC, USA) and AEs were coded using the Medical Dictionary for Regulatory Abbreviations (MedDRA) version 26.0.

The clinical investigation was exploratory, with no formal statistical hypothesis or sample size calculation. A total of 34 patients was deemed sufficient to meet the study objectives, allowing for an anticipated attrition rate of approximately 10%.

## 3. Results

### 3.1. Patient Disposition and Demographics

Thirty-four patients were screened and enrolled, and all completed the clinical investigation. The mean ± SD age of the patients was 39.7 ± 14.0 years (range: 20 to 73 years), and most were female (82.4%). All patients used soft contact lenses, and the mean daily wearing time of the contact lenses was 10.4 ± 2.3 h/day. The mean time since DED diagnosis was 27.9 ± 26.5 months. Most patients (76.5%) replaced their contact lenses monthly and 73.5% used a preserved contact lens solution (Table 1). As well as DED, most patients had myopia (82.4%), with other ocular medical histories including hypermetropia (14.7% of patients), astigmatism, and presbyopia (5.9% each). No concomitant ocular treatments were reported.

The daily dose regimen of T2769 reported at D36 ranged from 3 to 6 instillations daily, with 5 instillations/day being the most frequently used (16 patients, 47.1%), followed by 4 instillations (10 patients, 29.4%).

### 3.2. Performance Assessments

#### 3.2.1. Primary Performance Parameter

**Figure 1:**
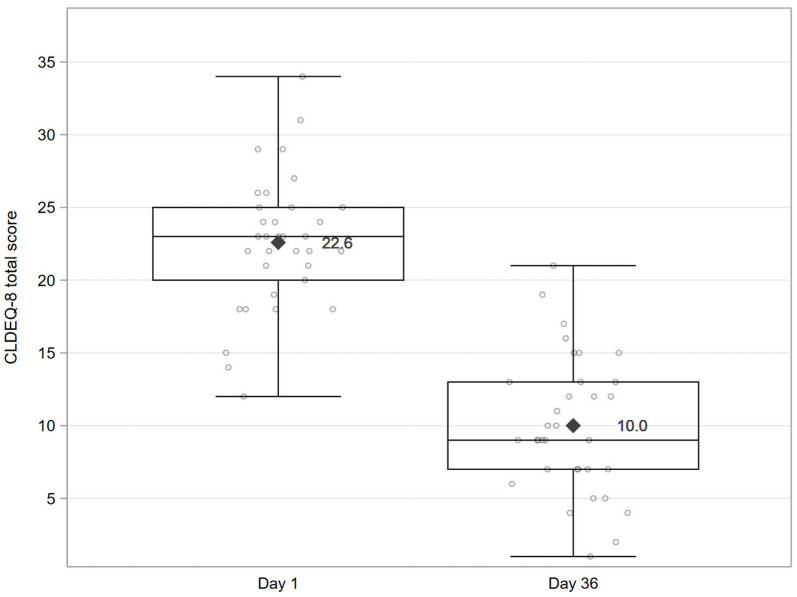
CLDEQ-8 total score at baseline (D1) and D36. Statistical comparisons are performed for changes from D1. The whiskers of the boxplot present the minimum and maximum values. Individual data points at each visit are shown as dots; mean values are indicated by a rhombus symbol, and whiskers represent the minimum and maximum values. Abbreviation: CLDEQ-8 = Contact Lens Dry Eye Questionnaire-8.

At baseline (D1), the mean comfort score, as measured by CLDEQ-8, was 22.6 ± 4.6. By D36, this score decreased to 10.0 ± 4.7, reflecting a mean change from baseline of −12.6 ± 5.0 [95% CI: −14.3; −10.8; *p* < 0.001]. See Figure 1.

#### 3.2.2. Other Performance Parameters

The mean ± SD CLDEQ-8 total score decreased from 22.6 ± 4.6 at baseline (D1) to 13.5 ± 5.2 at D15 (i.e., changes from baseline of −9.1 ± 4.5 [95% CI: −10.6; −7.5; *p* < 0.001]).

**Figure 2:**
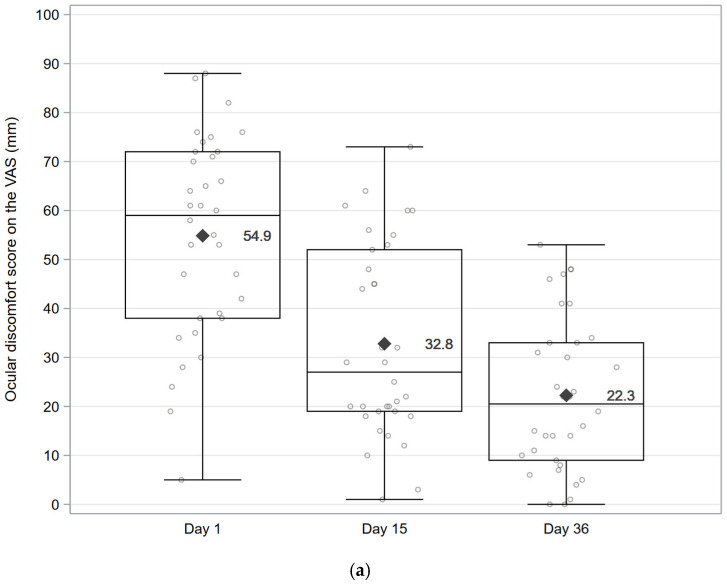

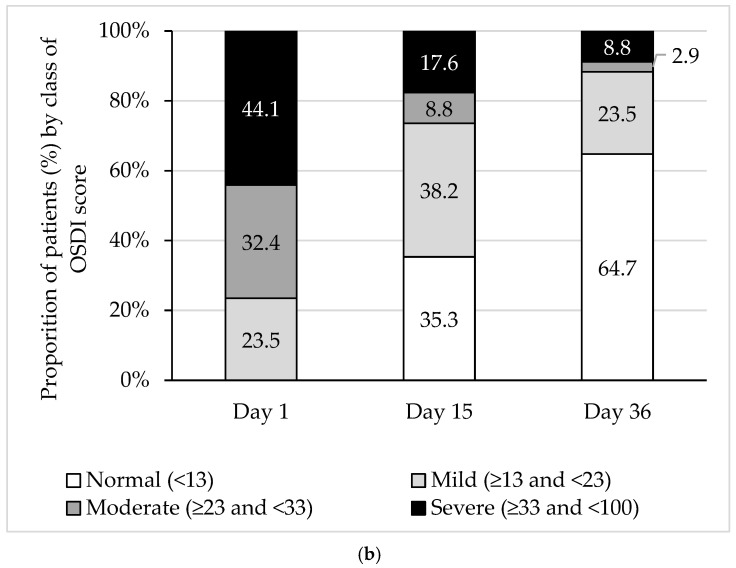

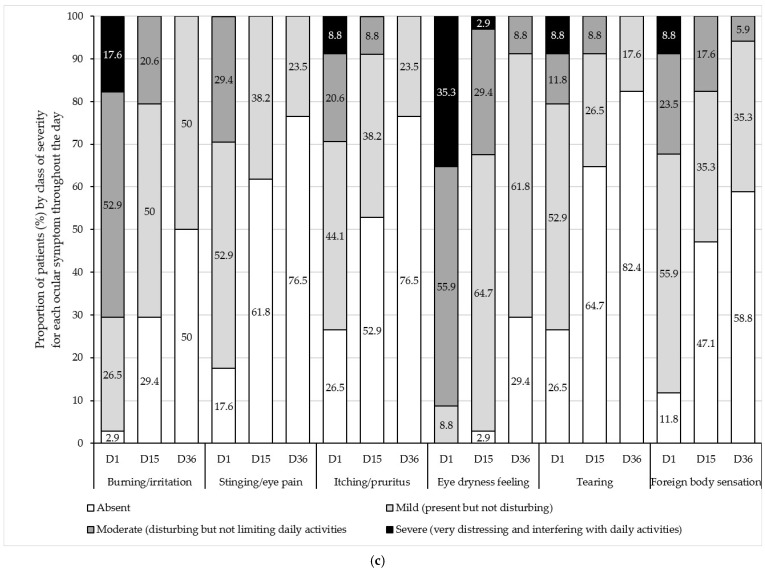
Ocular discomfort, ocular surface disease index (OSDI), and ocular symptoms by severity at baseline (D1), D15, and D36. (**a**) Ocular discomfort (VAS). Individual data points at each visit are shown as dots; mean values are indicated by a rhombus symbol, and whiskers represent the minimum and maximum values. (**b**) OSDI by class of severity. (**c**) Individual ocular symptoms throughout the day by class of severity. Abbreviations: D = Day; OSDI = Ocular Surface Disease Index; VAS = Visual Analogue Scale.

The mean ± SD ocular discomfort on the VAS decreased from 54.9 ± 20.6 mm at D1 to 32.8 ± 19.6 mm at D15 and 22.3 ± 15.8 mm at D36 (i.e., changes from the baseline of −22.1 ± 18.3 mm [95% CI: −28.4;−15.7; *p* < 0.001] and −32.6 ± 19.0 [95% CI: −39.2;−26.0; *p* < 0.001], respectively) (Figure 2a, Supplementary Table S1).

The mean ± SD OSDI score decreased from 36.9 ± 16.3 at D1 to 19.4 ± 14.4 at D15 and 12.7 ± 10.3 at D36, representing mean changes from baseline of −17.5 ± 11.9 [ 95% CI: −21.61; −13.33; *p* < 0.001) and −24.1 ± 12.6 [95% CI: −28.53; −19.76; *p* < 0.001], respectively. At D1, 76.5% of patients had an OSDI score ≥ 23 (moderate or severe disease), and none had an OSDI score < 13. By D36, 64.7% had a normal score (<13) and only 11.7% presented with moderate or severe disease (Figure 2b). Considering the threshold of ≥18 for DED diagnosis, all patients met the criteria for DED at D1 compared to 44.1% at D15 and 17.6% at D36.

Similarly, the mean ± SD total score for symptoms throughout the day decreased from 8.7 ± 3.1 at D1 to 4.3 ± 2.9 at D15 and 2.4 ± 1.9 at D36, with mean changes of −4.4 ± 2.4 [95% CI: −5.2; −3.5; *p* < 0.001] and −6.3 ± 3.2 [95% CI: −7.4; −5.2; *p* < 0.001], respectively. At D1, the most frequently reported moderate or severe symptoms were the feeling of eye dryness (91.2% of patients), followed by burning/irritation (70.5%), foreign body sensation (32.3%), stinging/eye pain and itching/pruritus (both 29.4%), and tearing (20.6%). By D36, no symptoms were reported by 82.4% of patients for tearing, 76.5% for itching/pruritus and stinging/eye pain, 58.8% for foreign body sensation, 50% for burning/irritation, and 29.4% for the feeling of eye dryness (Figure 2c).

**Figure 3:**
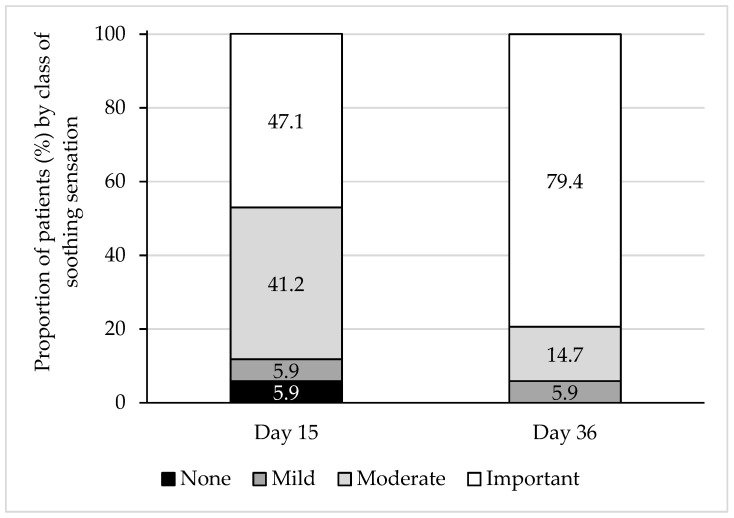
Soothing sensation within 5 min after instillation on D15 and D36.

The soothing sensation associated with T2769 use was considered important by 47.1% of patients at D15 and 79.4% of patients on D36 (Figure 3).

Daily contact lens wearing time. The median (min; max) daily contact lens wearing time remained constant throughout the clinical investigation in terms of hours/day (10.0 (6.0; 16.0) at D1, 10.6 (6.2; 16.0) at D15, and 11.0 (6.3; 15.3) at D36) and days/week (7.0 (5; 7) for all timepoints D1, D15, and D36).

**Figure 4:**
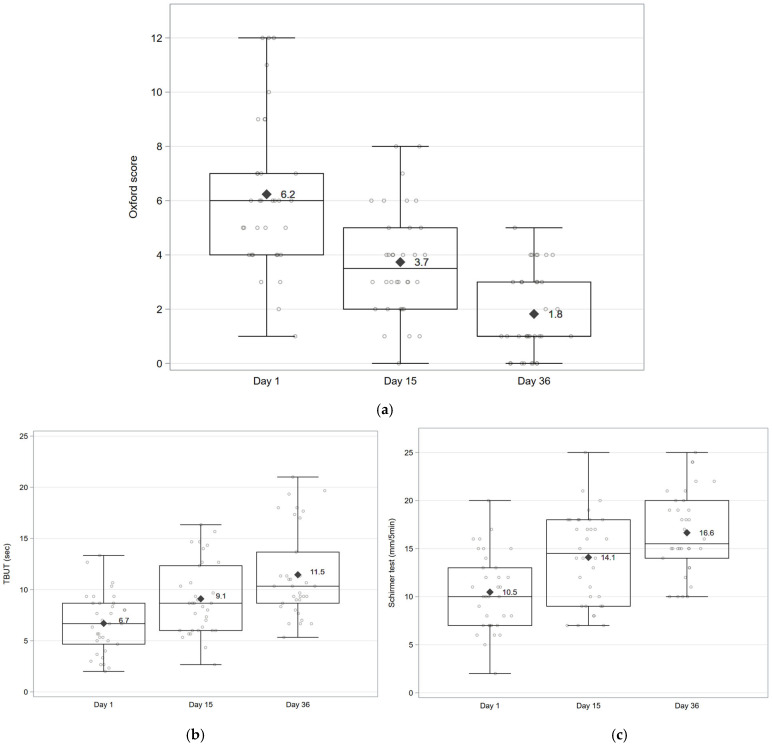
Global ocular surface staining (Oxford score), tear break-up time, and Schirmer test in the studied eye at baseline (D1), D15, and D36. The whiskers of the boxplot present the minimum and maximum values. (**a**) Global ocular surface staining (Oxford score 0–15). (**b**) Tear break-up time. (**c**) Schirmer test. Individual data points at each visit are shown as dots; mean values are indicated by a rhombus symbol, and whiskers represent the minimum and maximum values. Abbreviations: TBUT = Tear Break-Up Time.

Slit lamp examination. The mean ± SD change from baseline in the global ocular staining score decreased by −2.5 ± 1.8 at D15 [95% CI: −3.1; −1.9; *p* < 0.001] and −4.4 ± 2.2 at D36 [95% CI: −5.2; −3.6; *p* < 0.001] (Figure 4a). The mean ± SD TBUT (s) change from baseline was 2.4 ± 2.2 [95% CI: 1.6; 3.2; *p* < 0.001] at D15 and 4.8 ± 2.5 [95% CI: −3.9; 5.6; *p* < 0.001] at D36 (Figure 4b), and Schirmer test I values (mm/5 min) of 3.6 ± 2.6 at D15 [95% CI: 2.7; 4.5; *p* < 0.001] and 6.2 ± 3.5 at D36 [95% CI: 5.0; 7.4; *p* < 0.001]) (Figure 4c).

**Figure 5:**
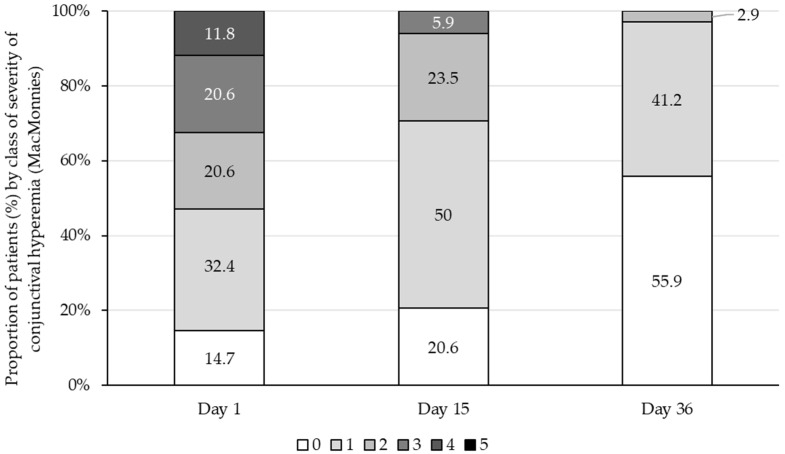
Conjunctival hyperaemia (McMonnies scale) in the studied eye at baseline (D1), D15, and D36. McMonnies 6-point scale: 0 = minimal conjunctival redness; 5 = maximal conjunctival redness. The performance of T2769 was considered by the investigators as very satisfactory or satisfactory for all patients on D15 and D36.

Regarding conjunctival hyperaemia, most patients (53.0%) had a score of ≥2 on D1 and 14.7% had no conjunctival hyperaemia (Figure 5). By D15, the percentage of patients with no conjunctival hyperaemia was 20.6%, which increased to 55.9% by D36. A total of 82.4% of patients showed an improvement at D36, with no patients reporting a worsening from baseline.

### 3.3. Safety

Ocular AEs were reported by two (5.9%) patients during the study. Of the four AEs reported, two were considered treatment-related, photophobia and instillation site discomfort, both reported by the same patient. One patient experienced a systemic AE (COVID-19) during the clinical investigation, which was not considered as treatment related. All AEs were non-serious and mild in severity.

Far BCVA remained constant during the clinical investigation (0.017 ± 0.045 LogMAR at D1 and 0.022 ± 0.051 LogMAR at D36).

Both the investigators and patients rated the overall ocular tolerance as satisfactory or very satisfactory on D15 and D36.

## 4. Discussion

This clinical investigation evaluated the ocular performance and safety of a new preservative-free ophthalmic formulation that combines sodium hyaluronate (0.15%), trehalose (3%), and NAAGA (2.45%) in adult contact lens wearers with dry eye symptoms. Eligible participants had frequent or intense symptoms of dry eyes requiring therapy, reflected by a baseline CLDEQ-8 score ≥ 12 and OSDI score ≥ 18. The CLDEQ-8, an eight-item validated questionnaire endorsed by the Tear Film and Ocular Surface Society (TFOS), is considered the most appropriate tool for identifying discomfort related to contact lens wear [23], while the OSDI is widely recognised for evaluating dry eye symptoms and ocular discomfort. Clinically meaningful improvements were observed in both measures (–12.6 ± 5.0 (CLDEQ-8) and −24.1 ± 12.6 (OSDI) from baseline to D36 (*p* < 0.001 for both)), demonstrating the beneficial effect of T2769 in this population where dry eyes and ocular discomfort are prevalent [18,19,24].

In contact lens wearers with dry eyes, clinical data from 0.15% sodium hyaluronate alone (SH; HYABAK^®^, Laboratoires Théa, Clermont-Ferrand, France) and 0.15% SH/3% trehalose (Thealoz^®^ Duo) reported improvements in symptoms and signs of DED and ocular comfort after D28 and D84 [14,15], with some of them, including TBUT and Van Bijsterveld conjunctival staining, remaining clinically and statistically non-significant [15]. In contrast, the magnitude of improvements with T2769 was greater in both subjective and objective endpoints, including CLDEQ-8, OSDI, VAS discomfort, TBUT, Schirmer I, and Oxford staining, despite a shorter follow up (D15 and D36). For instance, the mean changes from baseline in CLDEQ-8 and OSDI scores after 84 days of Thealoz^®^ Duo were −6.5 ± 5.0 and −14.1 ± 13.6, respectively, compared with −12.6 and −24.1, respectively, for T2769 after 36 days). These findings suggest that T2769 may provide a more rapid and robust clinical benefit in contact lens wearers. This enhanced effect may be linked to the inclusion of NAAGA, a naturally occurring amino-conjugated dipeptide with several anti-inflammatory properties [25]. NAAGA supports ocular surface homeostasis by inhibiting mast cells’ histamine release, activation of the complement system, leukocyte adhesion and recruitment to inflammatory sites, and the expression of adhesion molecules on granulocytes and endothelial cells [13,26,27]. In a clinical study in DED patients, NAAGA significantly reduced HLA-DR (Human Leukocyte Antigen-DR isotype, a cell surface antigen involved in triggering the immune response) expression and inflammatory marker, compared to placebo [13]. Naabak^®^ (NAAGA 4.9%) is a well-established anti-inflammatory agent used for allergic conjunctivitis with treatment periods longer than 30 days. It has been marketed for more than 40 years in therapeutics and showed a favourable safety profile. NAAGA is included in the T2769 eye drop formulation to support the effects of sodium hyaluronate and trehalose [27,28,29,30]. This combination may result in faster relief from inflammatory-related symptoms and signs, including conjunctival hyperaemia [13,16,31], consistent with TFOS recommendations for the early initiation of topical anti-inflammatory agents in DED management. Therefore, the components of the formulation are thought to contribute to the improvement in pre-lens tear film stability. Sodium hyaluronate enhances hydration and lubrication through its hygroscopic and viscoelastic properties, while trehalose provides osmoprotection and preserves epithelial membrane components under desiccating stress. The ancillary medicinal substance NAAGA may limit the inflammatory process associated with dry eyes and lens wear.

A previous clinical investigation in Tunisia demonstrated the clinical benefits of the T2769 formulation [16] in patients with moderate to severe DED, but who did not wear contact lenses. The results of the present study in contact lens wearers are consistent with those findings and confirm the clinical performance of T2769 in a European population. Performance outcomes improved rapidly by D15 of treatment and continued to improve through D36. The safety profile observed in contact lens wearers was also comparable to that observed in contact lens non-wearers with DED [16]. This favourable safety profile, supported by the well-established tolerability of sodium hyaluronate, trehalose, and NAAGA [12,32,33,34,35], may enhance patient adherence. In addition, previous testing of T2769 with hydrogel, silicon hydrogel, and rigid gas-permeable contact lenses, confirmed excellent biocompatibility. Relief of contact lens discomfort with T2769 treatment may help reduce the discontinuation of contact lens use in individuals experiencing dry eye symptoms.

DED is the most common complication after refractive surgery such as LASIK, involving corneal nerve disruption, decreased reflex lacrimal secretion, and tear film instability [36]. Artificial tears are a common perioperative treatment that can provide symptomatic relief and promote early restoration of the tear film stability and ocular surface damage. In our study, T2769 showed a soothing sensation within 5 min of instillation and demonstrated rapid improvements in TBUT, Schirmer I, and ocular surface staining by D15, as well as favourable contact lens biocompatibility. This suggests that T2769 may be an option to support tear film restoration in the perioperative period.

A limitation of this investigation was that all participants were soft-lens-wearers, although both soft and rigid gas-permeable lenses were permitted. This reflects current clinical practice, where rigid gas-permeable lenses represent only a small proportion of lens fittings. In Bulgaria (where this investigation was conducted), rigid gas-permeable lenses prescribing rates are approximately 10.5%, consistent with trends across Europe [37]. Future studies specifically including rigid gas-permeable wearers may be needed to confirm whether the observed benefits of the NAAGA-based formulation extend to this group.

Another limitation is the small sample size and lack of control group, as this study was designed to provide descriptive rather than hypothesis-driven data. However, the consistency of these findings with those from contact lens non-wearers [16], and the greater objective improvements observed with the NAAGA-containing formulation compared with Thealoz^®^ Duo [13,15], support its clinical benefit in this population. Given the single-arm design, a placebo effect or natural course of symptom resolution over time may have contributed to the observed improvements. While NAAGA may provide additional anti-inflammatory support, its specific contribution to the observed clinical benefits cannot be shown in this study.

In conclusion, this clinical investigation demonstrated the clinical benefits of T2769 in managing dry eyes in contact lens wearers, consistent with findings previously observed in contact lens non-users with DED. These results suggest that T2769 could significantly improve both symptoms and signs, thereby enhancing quality of life for contact lenses wearers.

## Figures and Tables

**Table 1:** Demographic and dry eye characteristics at baseline (FAS).

	Total (N = 34)
Sex (n, %)	
Female	28 (82.4%)
Male	6 (17.6%)
Age (years)	
Mean ± SD	39.7 ± 14.0
Min, max	20, 73
Age classes (n, %)	
<65 years	33 (97.1%)
≥65 and <85 years	1 (2.9%)
≥85 years	0
Contact lens history	
Contact lens type (n, %)	
Soft	34 (100.0%)
Replacement modality (n, %)	
Daily	3 (8.8%)
Weekly	0
Biweekly	1 (2.9%)
Monthly	26 (76.5%)
Every 3 months	3 (8.8%)
Every 6 months	1 (2.9%)
Yearly	0
Contact lens solution type ^a^ (n, %)	
Preserved	25 (73.5%)
Not preserved	8 (23.5%)
None	1 (2.9%) ^b^
Contact lens wearing time (mean ± SD)	
Hours/day	10.44 ± 2.31
Days/week	6.4 ± 0.9
Ocular DED history	
Time since DED diagnosis (months) (mean ± SD)	27.9 ± 26.5
Min, max	0, 120

DED = Dry Eye Disease; FAS = Full Analysis Set; min, minimum; max, maximum; SD = Standard Deviation. ^a^ Two patients with daily contact lenses used a preserved solution to rinse the lenses during the day. ^b^ The patient wore daily disposable contact lenses, without cleaning or disinfecting solution.

## Data Availability

The data presented in this study are openly available in ClinicalTrials.gov, reference number NCT05931861. Further inquiries can be directed to the corresponding author.

## Supplementary Materials

The following supporting information can be downloaded at: https://www.mdpi.com/article/10.3390/vision10010001/s1, Table S1. Individual VAS discomfort scores (0–100 mm scale) at D1, D15, and D36.

## Abbreviations

The following abbreviations are used in this manuscript:

EUDES	European Dry Eye Society
IRB	Institutional Review Board
QoL	Quality of Life
AE	Adverse Event
BCVA	Best-Corrected Visual Acuity
CI	Confidence Interval
CLD	Contact Lens Discomfort
CLDEQ-8	Contact Lens Dry Eye Questionnaire-8
EVER	European Association for Vision and Eye Research
FAS	Full Analysis Set
GCP	Good Clinical Practice
IOL	Intraocular Lens
N/A	Not Applicable
NAAGA	N-Acetylaspartylglutamic Acid
OSDI	Ocular Surface Disease Index
PP	Per Protocol
SAE	Serious Adverse Event
SD	Standard Deviation
SE	Standard Error
SOP	Standard Operating Procedure
TBUT	Tear Break-Up Time
TFOS	Tear Film and Ocular Surface Society
VA	Visual Analogue Scale
