# Supplementary material for: N-Acetyl-Aspartyl Glutamic Acid (NAAGA)-Based Eye Drops for Contact Lens Wearers with Dry Eye Symptoms and Discomfort
Source: Vision (Basel). 2025 Dec 22;10(1):1. doi: 10.3390/vision10010001 (PMC12821722; doi:10.3390/vision10010001)
Supplement: Supplementary file 1 [file vision-10-00001-s001.zip › vision-3960865-supplementary.pdf]

Patient	D1	D15	D36
P1	76	55	41
P2	88	73	53
P3	74	53	31
P4	61	44	34
P5	65	45	33
P6	71	61	48
P7	75	64	46
P8	66	60	33
P9	70	60	47
P10	60	52	48
P11	24	32	28
P12	35	20	0
P13	38	1	1
P14	28	20	0
P15	19	18	8
P16	72	10	4
P17	76	21	6
P18	39	29	24
P19	82	56	30
P20	64	15	23
P21	61	14	9
P22	87	12	14
P23	34	19	7
P24	30	25	15
P25	58	32	22
P26	42	20	11
P27	47	18	14
P28	72	45	19
P29	55	48	14
P30	53	20	5
P31	53	22	16
P32	47	29	22
P33	5	3	10
P34	38	19	41

Supplementary Table S1. Individual VAS discomfort scores (0–100 mm scale) at D1, D15, and D36.
